# Mansoor's Self‐Report Tool for Cardiovascular Risk Assessment predicts adverse in‐hospital events in patients with pulmonary embolism

**DOI:** 10.1111/crj.13571

**Published:** 2022-12-21

**Authors:** Karsten Keller, Volker H. Schmitt, Mir A. Ostad, Thomas Münzel, Lukas Hobohm, Christine Espinola‐Klein

**Affiliations:** ^1^ Department of Cardiology, Cardiology I University Medical Center Mainz (Johannes Gutenberg‐University Mainz) Mainz Germany; ^2^ Center for Thrombosis and Hemostasis (CTH) University Medical Center Mainz (Johannes Gutenberg‐University Mainz) Mainz Germany; ^3^ Medical Clinic VII, Department of Sports Medicine University Hospital Heidelberg Heidelberg Germany; ^4^ German Center for Cardiovascular Research (DZHK), Partner Site Rhine Main Mainz Germany

**Keywords:** bleeding, mortality, pulmonary embolism, risk stratification

## Abstract

**Background:**

Pulmonary embolism (PE) is a life‐threatening acute disease accompanied by high morbidity and mortality. Regarding hospitalizations of patients with PE, risk stratification of these patients is crucial. Thus, risk stratification tools like risk scores are of key interest.

**Methods:**

The nationwide German inpatient sample of the years 2005–2018 was used for this present analysis. Hospitalized PE patients were stratified according to Mansoor's Self‐Report Tool for Cardiovascular Risk Assessment class, and the performance of this score was evaluated to predict adverse in‐hospital events.

**Results:**

Overall, 1 174 196 hospitalizations of PE patients (53.5% females; 56.4% ≥70 years) were registered in Germany between 2005 and 2018. According to the Mansoor's self‐report tool for cardiovascular risk assessment, 346 126 (29.5%) PE patients were classified as high risk.

Higher Mansoor's Self‐Report Tool for Cardiovascular Risk Assessment class was predictive for in‐hospital death (OR 1.129 [95%CI 1.117–1.141], *P* < 0.001), shock (OR 1.117 [95%CI 1.095–1.140], *P* < 0.001), cardiopulmonary resuscitation (OR 1.109 [95%CI 1.092–1.126], *P* < 0.001), right ventricular dysfunction (OR 1.039 [95%CI 1.030–1.048], *P* < 0.001), intracerebral bleeding (OR 1.316 [95%CI 1.275–1.358], *P* < 0.001), and gastro‐intestinal bleeding (OR 1.316 [95%CI 1.275–1.358], *P* < 0.001). Systemic thrombolysis was not associated with lower in‐hospital mortality in high‐risk class (OR 5.139 [95%CI 4.961–5.323], *P* < 0.001).

**Conclusions:**

Prognostic performance of the Mansoor's Self‐Report Tool for Cardiovascular Risk Assessment for risk stratification of PE patients was poor and not able to identify those PE patients, who might benefit from systemic thrombolysis. However, the Mansoor's Self‐Report Tool for Cardiovascular Risk Assessment was moderately helpful to identify PE patients at higher risk for bleeding events.

## Graphical abstract

Prognostic performance of the Mansoor's Self‐Report Tool for Cardiovascular Risk Assessment to identify PE patients with higher risk for in‐hospital death and adverse events during in‐hospital stay.

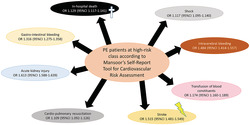

**Abbreviations**
CI: confidence interval
DRG: Diagnosis Related Groups
DVT: deep venous thrombosis and/or thrombophlebitis of the leg veins
ICD: International Classification of Diseases and Related Health Problems
IQR: inter‐quartile range
OPS: diagnostic, surgery and procedures codes (Operationen‐ und Prozedurenschlüssel)
OR: odds ratio
PE: pulmonary embolism
RDC: research data center
VTE: venous thromboembolism

## 1 INTRODUCTION

Pulmonary embolism (PE) is a life‐threatening acute disease accompanied by high morbidity and mortality.^1^, ^2^, ^3^, ^4^, ^5^, ^6^, ^7^, ^8^ In patients with acute PE, it is well established that right ventricular dysfunction (RVD) during the acute phase (assessed by echocardiography or computed tomography), myocardial injury, and hemodynamic status are important predictors of adverse in‐hospital outcomes.^2^, ^3^, ^4^, ^6^, ^7^, ^8^, ^9^, ^10^, ^11^, ^12^, ^13^, ^14^, ^15^, ^16^, ^17^ In addition, several approaches with different scores were tested, and some of them established for risk stratification of PE patients in order to identify patients at higher risk to develop adverse events and especially those, who should be treated with reperfusion treatments.^6^, ^7^, ^18^, ^19^, ^20^, ^21^ The recently published Mansoor's Self‐Report Tool for Cardiovascular Risk Assessment is an interesting and novel simple risk score to predict cardiovascular events in adults and might also be helpful for risk stratification in PE patients.^22^

## 2 MATERIAL AND METHODS

### 2.1 Data source

The data of the German nationwide inpatient sample statistics (diagnosis related groups [DRG] statistic) were used for this present study analysis (source: Research Data Center [RDC] of the Federal Statistical Office and the Statistical Offices of the federal states, DRG Statistics 2005–2018, own calculations). The diagnostic and treatment data from all patients hospitalized in German hospitals, which were processed according to the DRG system, were gathered/collected by the Federal Statistical Office of Germany (Statistisches Bundesamt, Wiesbaden, Germany). The hospitals have to code the diagnoses according the ICD‐10‐GM (International Classification of Diseases, 10th Revision with German Modification) and diagnostical, surgical, and interventional procedures according OPS codes (surgery, diagnostic, and procedures codes [Operationen‐ und Prozedurenschlüssel]) to get their reimbursement of their costs and the German nationwide inpatient sample comprises all hospitalized PE patients (ICD‐code I26) of the investigated years 2005–2018.

PE patients were stratified according the Mansoor's Self‐Report Tool for Cardiovascular Risk Assessment classes (Table 1) in patients with low‐risk (≤5 points) or high‐risk (>5 points) status.^22^ One modification was done regarding the present calculation of the Mansoor's Self‐Report Tool for Cardiovascular Risk Assessment: Instead of family history of myocardial infarction, myocardial infarction in patient's medical history was assessed as one of the parameters of the score.

**TABLE 1:** Parameters and scoring of the modified Mansoor's Self‐Report Tool for Cardiovascular Risk Assessment score^22^

Parameters	Score (points)
Age groups with scoring:	
>59 and <70 years	+2 points
>69 years	+3 points
Male gender	+2 points
Current smoking	+2 points
Diabetes mellitus	+3 points
Arterial hypertension	+2 points
Myocardial infarction in medical history	+1 points
Categorization of risk classes: low risk class (≤5 points) high risk class (>5 points)	

### 2.2 Study endpoints and in‐hospital adverse events

The study outcomes comprise all‐cause in‐hospital death, cardiopulmonary resuscitation (CPR; OPS code 8‐77), shock (ICD code R57), stroke (ischemic or hemorrhagic) (ICD codes I61‐I64), acute kidney injury (ICD code N17), serious bleeding events such as intracerebral bleeding (ICD code I61), gastro‐intestinal bleeding (ICD codes K920‐K922), and necessity of transfusion of blood components (OPS code 8‐800). In addition, we defined a composite outcome of all‐cause in‐hospital death, CPR, shock, stroke, intracerebral bleeding, gastro‐intestinal bleeding, necessity of transfusion of blood components, and/or acute kidney injury.

### 2.3 Definitions

Obesity was defined as a body mass index ≥30 kg/m^2^ as recommended by the WHO (World Health Organization). Stroke comprised both stroke entities: ischemic and haemorrhagic stroke. Hemodynamically unstable PE was defined as PE patients with shock or cardio‐pulmonary resuscitation.

### 2.4 Ethical aspects

Since in our present study the investigators had not a direct access to data of individual patients and the cumulative results were provided by the Research data center [RDC] of the Federal Statistical Office and the Statistical Offices of the federal states, approval by an ethics committee and informed consent were not required, in accordance with the German law.

### 2.5 Statistical methods

Descriptive statistics for relevant patient characteristics' comparisons of PE patients of the different Mansoor's Self‐Report Tool for Cardiovascular Risk assessment score classes are provided as median and interquartile range (IQR) or absolute numbers and corresponding percentages. We tested continuous variables of the compared study groups with the Mann–Whitney‐U test and categorical variables using the Fisher's exact or the chi^2^ test (as appropriate).

Temporal trends with regard to the distribution of the different Mansoor's Self‐Report Tool for Cardiovascular Risk Assessment score classes stratified for the treatment year as well as for patients' age decades were calculated annually, and linear regressions were performed to evaluate and investigate trends over time. The results are presented as beta (β) and corresponding 95% confidence intervals (CI).

Univariable logistic regression models were computed in order to test associations of a higher class of the calculated Mansoor's Self‐Report Tool for Cardiovascular Risk Assessment score and different study endpoints. Results are presented as odds ratio (OR) and corresponding 95%CI. The software SPSS® (version 20.0; SPSS Inc., Chicago, Illinois) was used for computerized statistical analysis. *P* values of <0.05 (two‐sided) were considered to be statistically significant.

## 3 RESULTS

**FIGURE 1:**
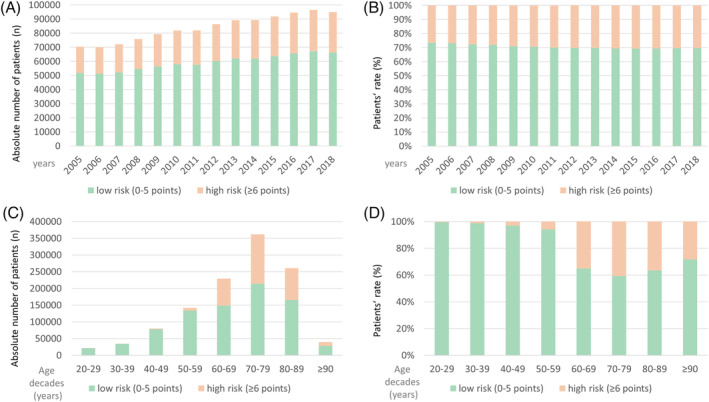
Temporal trends on hospitalized PE patients in Germany 2005–2018: absolute and relative numbers stratified for Mansoor's Self‐Report Tool for Cardiovascular Risk Assessment risk classes. (A) Absolute numbers of hospitalized PE patients 2005–2018 stratified for Mansoor's Self‐Report Tool for Cardiovascular Risk Assessment risk classes. (B) Hospitalized PE patients stratified for Mansoor's Self‐Report Tool for Cardiovascular Risk Assessment risk classes (proportions) 2005–2018. (C) Absolute numbers of hospitalized PE patients 2005–2018 stratified for Mansoor's Self‐Report Tool for Cardiovascular Risk Assessment risk classes in the different age‐decades. (D) Hospitalized PE patients stratified for Mansoor's Self‐Report Tool for Cardiovascular Risk Assessment risk classes (proportions) 2005–2018 in the different age‐decades

Overall, 1 174 196 hospitalizations of PE patients (53.5% females; 56.4% ≥70 years) were registered in Germany between 2005 and 2018 and were included in the present study. The total number of hospitalizations of PE patients increased from 2005 to 2018 (Figure 1A). Largest number of hospitalizations was in the 8th decade of life (Figure 1C).

According to the Mansoor's self‐report tool for cardiovascular risk assessment, in total, 828 070 (70.5%) of the hospitalized PE patients were classified as low risk, 346 126 (29.5%) as high risk (Table 2).

**TABLE 2:** Hospitalized 1 174 196 PE patients in Germany 2005–2018 stratified according to Mansoor's Self‐Report Tool for Cardiovascular Risk Assessment

	Low risk (0–5 points) (*n* = 828 070 [70.5%])	High risk (≥6 points) (*n* = 346 126 [29.5%])	*P* value
Age (IQR) in years	69.0 (55.0–79.0)	75.0 (69.0–81.0)	<0.001
Age ≥70 years	407 767 (49.2%)	254 331 (73.5%)	<0.001
Female sex^a^	519 062 (62.7%)	108 558 (31.4%)	<0.001
Length of in‐hospital stay (IQR) in days	9.0 (5.0–15.0)	11.0 (6.0–18.0)	<0.001
Obesity	71 023 (8.6%)	41 471 (12.0%)	<0.001
Hyperlipidemia	71 927 (8.7%)	70 760 (20.4%)	<0.001
Comrobidities			
Coronary artery disease	72 638 (8.8%)	88 750 (25.6%)	<0.001
Peripheral artery disease	1347 (0.2%)	1617 (0.5%)	<0.001
Atrial fibrillation/flutter	104 893 (12.7%)	73 946 (21.4%)	<0.001
Cancer	165 468 (20.0%)	71 030 (20.5%)	<0.001
Chronic obstructive pulmonary disease	70 108 (8.5%)	50 479 (14.6%)	<0.001
Essential arterial hypertension	243 866 (29.4%)	265 461 (76.7%)	<0.001
Diabetes mellitus	23 991 (2.9%)	195 559 (56.5%)	<0.001
Renal insufficiency (GFR < 60 ml/min/1,73 m^2^)	66 036 (8.0%)	59 398 (17.2%)	<0.001
Right ventricular dysfunction, sPESI and deep venous thrombosis and/or thrombophlebitis			
Hemodynamically unstable PE	71 937 (8.7%)	33 422 (9.6%)	<0.001
Right ventricular dysfunction	234 223 (28.3%)	100 599 (29.1%)	<0.001
sPESI – high risk class (score ≥1)	473 122 (57.0%)	256 959 (74.0%)	<0.001
Deep venous thrombosis and/or thrombophlebitis	303 404 (36.6%)	117 009 (33.8%)	<0.001
Reperfusion treatment			
Systemic thrombolysis	35 173 (4.2%)	13 985 (4.0%)	<0.001
Surgical embolectomy	1340 (0.2%)	441 (0.1%)	<0.001
In‐hospital death and adverse events during hospitalization			
Composite outcome	226 229 (27.3%)	110 668 (31.9%)	<0.001
In‐hospital death	126 474 (15.3%)	58 543 (16.9%)	<0.001
Shock	31 156 (3.8%)	14 482 (4.2%)	<0.001
Cardio‐pulmonary resuscitation	53 424 (6.5%)	24 590 (7.1%)	<0.001
Stroke (ischemic or hemorrhagic)	20 421 (2.5%)	12 769 (3.7%)	<0.001
Acute kidney injury	42 113 (5.1%)	27 537 (8.0%)	<0.001
Intracerebral bleeding	4381 (0.5%)	2710 (0.8%)	<0.001
Gastro‐intestinal bleeding	11 100 (1.3%)	6081 (1.8%)	<0.001
Transfusion of blood constituents	91 741 (11.1%)	44 184 (12.8%)	<0.001

^a^ Information available for analysis in 1 174 135 patients.

The proportion of high‐risk class categorization increased slightly over time from 2005 to the year 2018 (β 0.229 [95%CI 0.213 to 0.245], *P* < 0.001), when analyzed with linear regression models (Figure 1B), and as expected, with growing age (β 0.876 [95%CI 0.870 to 0.882], *P* < 0.001) (Figure 1D).

As expected, PE patients classified as low risk group were younger (69.0 vs. 75.0 years, *P* < 0.001) and had less often cardiovascular risk factors and comorbidities, COPD as well as renal failure in comparison to the high‐risk class group (Table 2). While deep venous thrombosis and/or thrombophlebitis of the superficial veins were more prevalent in patients classified low‐risk according to the Mansoor's self‐report tool for cardiovascular risk assessment, haemodynamically unstable PE, sPESI–high risk class (score ≥1), and RVD were more frequent in the high‐risk class of this score (Table 2).

As shown in Table 2, in‐hospital death and all other investigated adverse events during hospitalization were more frequent in patients classified high‐risk according to the Mansoor's self‐report tool for cardiovascular risk assessment. In‐hospital mortality was 16.9% in the high‐risk and 15.3% in the low‐risk group according to the Mansoor's self‐report tool for cardiovascular risk assessment. The investigated composite outcome was also more frequently observed in high‐risk versus low‐risk group (31.9% vs. 27.3%, *P* < 0.001) (Table 2).

A high‐risk Mansoor's Self‐Report Tool for Cardiovascular Risk Assessment class was predictive for in‐hospital death (OR 1.129 [95%CI 1.117–1.141], *P* < 0.001), the composite outcome (OR 1.249 [95%CI 1.239–1.260], *P* < 0.001), and signs of decompensated status of PE such as shock (OR 1.117 [95%CI 1.095–1.140], *P* < 0.001), cardiopulmonary resuscitation (OR 1.109 [95%CI 1.092–1.126], *P* < 0.001), and RVD (OR 1.039 [95%CI 1.030–1.048], *P* < 0.001) (Table 3). High risk class was also associated with bleeding events like intracerebral bleeding (OR 1.316 [95%CI 1.275–1.358], *P* < 0.001), gastro‐intestinal bleeding (OR 1.316 [95%CI 1.275–1.358], *P* < 0.001), and need for transfusion of blood constituents (OR 1.174 [95%CI 1.160–1.189], *P* < 0.001) (Table 3).

**TABLE 3:** Impact of Mansoor's Self‐Report Tool for Cardiovascular Risk Assessment classes on in‐hospital death and adverse events during in‐hospital stay in patients with pulmonary embolism (univariable logistic regression model)

	Univariable regression model	
	OR (95% CI)	*P* value
Composite outcome	1.249 (1.239–1.260)	<0.001
In‐hospital death	1.129 (1.117–1.141)	<0.001
Shock	1.117 (1.095–1.140)	<0.001
Cardio‐pulmonary resuscitation	1.109 (1.092–1.126)	<0.001
Stroke (ischemic or hemorrhagic)	1.515 (1.481–1.549)	<0.001
DVT	0.883 (0.876–0.891)	<0.001
Right ventricular dysfunction	1.039 (1.030–1.048)	<0.001
Acute kidney injury	1.613 (1.588–1.639)	<0.001
Intracerebral bleeding	1.484 (1.414–1.557)	<0.001
Gastro‐intestinal bleeding	1.316 (1.275–1.358)	<0.001
Transfusion of blood constituents	1.174 (1.160–1.189)	<0.001
Pneumonia	1.049 (1.039–1.058)	<0.001

Systemic thrombolysis was not associated with lower in‐hospital mortality in low‐risk class (univariate: OR 5.026 [95%CI 4.917–5.138], *P* < 0.001; multivariate adjusted for age and sex: OR 6.104 [95%CI 5.965–6.246], *P* < 0.001) but also not associated to lower mortality in high‐risk class (univariate: OR 4.629 [95%CI 4.472–4.792], *P* < 0.001; multivariate adjusted for age and sex: OR 5.139 [95%CI 4.961–5.323], *P* < 0.001) according to the Mansoor's self‐report tool for cardiovascular risk assessment.

When comparing the Mansoor's self‐report tool for cardiovascular risk assessment to the sPESI, sPESI was more suitable to predict in‐hospital death (OR 2.918 [95%CI 2.883–2.954], *P* < 0.001) as well as the composite outcome (OR 3.411 [95%CI 3.378–3.443], *P* < 0.001).

## 4 DISCUSSION

An acute pulmonary embolism (PE) is a cardiovascular emergency case with high morbidity and mortality.^1^, ^2^, ^3^, ^4^, ^5^, ^6^, ^7^, ^8^ Adverse short‐term outcome of acute PE is closely related to initial hemodynamic status, myocardial injury, and RVD.^2^, ^3^, ^4^, ^6^, ^7^, ^8^, ^9^, ^10^, ^11^, ^12^, ^13^, ^14^, ^15^, ^16^, ^17^ In addition, several scores were developed, tested, and established for risk stratification of PE patients in order to identify patients at higher risk for adverse events.^6^, ^18^, ^19^, ^20^ The recently published Mansoor's Self‐Report Tool for Cardiovascular Risk Assessment is an interesting and novel simple risk score to predict cardiovascular events and might also be helpful for risk stratification in PE patients.^22^

The main results of our study can be summarized as follows:

- The total number of hospitalizations due to PE in both Mansoor's Self‐Report Tool for Cardiovascular Risk Assessment risk classes increased over time.
- The hospitalizations increased with patients' age and were largest in the 8th decade of life.
- According to the Mansoor's self‐report tool for cardiovascular risk assessment, 1/3 of the patients were classified as high‐risk status.
- Higher Mansoor's Self‐Report Tool for Cardiovascular Risk Assessment class was predictive for in‐hospital mortality, the composite outcome, decompensated status and bleeding events in PE patients, but prognostic performance was substantially weaker in comparison to the sPESI.
- The Mansoor's Self‐Report Tool for Cardiovascular Risk Assessment risk classes were not able to identify those patients at high‐risk, who benefit from systemic thrombolysis.

Short‐term and particularly in‐hospital PE‐related mortality risk is primarily based on the patient's clinical status at presentation, the comorbidities, and the treatment.^6^, ^7^, ^21^, ^23^, ^24^, ^25^ Whereas hemodynamic instability in PE patients is a life‐threatening condition with highest risk to die that requires immediate reperfusion treatment,^6^, ^7^, ^26^ regarding hemodynamically stable PE or VTE (with absence of cardiac arrest or cardiogenic shock), it is crucial to be further stratified by advanced risk stratification strategies.^6^, ^7^, ^21^, ^25^, ^27^

Current international guidelines recommend treatment with anticoagulant therapy for PE of at least 3 months^6^, ^28^ aiming to treat the current venous thromboembolic clot of PE and/or deep venous thrombosis (dissolve the thrombus and/or embolus) and to prevent sequalae of the venous thromboembolism (VTE) event as well as recurrent VTE events.^6^, ^21^, ^28^, ^29^, ^30^, ^31^, ^32^, ^33^

Despite these important beneficial effects of anticoagulant treatment in VTE patients, anticoagulation also imposes with an increased risk for bleeding events.^6^, ^21^, ^29^ Bleeding events are the main adverse outcome and potentially life‐threatening complications of this therapy.^21^, ^34^, ^35^ For therapy planning of PE and VTE patients as well as the monitoring of these patients, it is of outstanding interest to assess individual bleeding risk,^6^, ^21^, ^29^ although this assessment is challenging.^21^, ^36^, ^37^

Thus, risk stratification during the acute phase of PE in patients with PE comprises the evaluation of the risk for adverse events caused by the acute PE event, the aggravation of consisting comorbidities, and treatment complications.^21^, ^36^, ^37^

Although several risk assessment models and scores have been developed in the past years and decades, most of them are complex and connected with time‐consuming assessment.^21^ Thus, the Mansoor's Self‐Report Tool for Cardiovascular Risk Assessment is an exception, since this score can be easily computed out of the patient characteristics (age, sex, and existing comorbidities) at the admission of the patient.^22^

Our results demonstrate that the Mansoor's Self‐Report Tool for Cardiovascular Risk Assessment is moderate and in part helpful but in comparison to the sPESI a weak risk stratification tool to predict adverse in‐hospital events under actual treatment regimens recommended by the current and recent guidelines (including the non‐vitamin K antagonist oral anticoagulants [NOACs]), showing once again that also comorbidities are influencing the outcome of PE patients.^6^, ^12^, ^25^, ^28^

Higher Mansoor's Self‐Report Tool for Cardiovascular Risk Assessment class was predictive for in‐hospital death as well as the composite outcome. The in‐hospital mortality was 1.13‐fold increased, if a high‐risk class according to the Mansoor's Self‐Report Tool for Cardiovascular Risk Assessment was present in the PE patients in comparison to the low‐risk group. Nevertheless, the differentiation between high‐ and low‐risk class regarding in‐hospital mortality was accompanied by an only small difference regarding the in‐hospital mortality of 1.6% (16.9% vs. 15.3%). In comparison to the Mansoor's Self‐Report Tool for Cardiovascular Risk Assessment, the high‐risk class regarding the sPESI was substantially stronger associated with in‐hospital death as well as the composite outcome. Importantly for risk stratification and planning of aggressive reperfusion treatments such as systemic thrombolysis and surgical embolectomy, high‐risk class according to the Mansoor's Self‐Report Tool for Cardiovascular Risk Assessment demonstrated regarding higher risk of decompensated and/or hemodynamically unstable PE such as 1.12‐fold risk of shock, 1.11‐fold risk of CPR, and 1.04‐fold risk of RVD, even though these associations were not strong.

In line with these small predictive values regarding decompensated and/or hemodynamically unstable PE as well as in‐hospital mortality, the Mansoor's Self‐Report Tool for Cardiovascular Risk Assessment was not able to identify those patients at high‐risk, who benefit from systemic thrombolysis. This finding underlines once again the weakness of the Mansoor's Self‐Report Tool for Cardiovascular Risk Assessment regarding the prognostic performance in PE patients, since it is of outstanding importance to identify patients, who benefit from reperfusion treatments such as systemic thrombolysis in the light of the complication rates of these aggressive treatments.^6^, ^7^, ^27^, ^31^ Two recently published projects of our group revealed that unselected normotensive PE patients did not benefit from systemic thrombolysis,^7^ whereas selected normotensive PE patients with incident decompensation such as e.g. normotensive PE patients with syncope might benefit from this aggressive treatment strategy.^38^ In summary, the Mansoor's Self‐Report Tool for Cardiovascular Risk Assessment was not helpful to identify those PE patients with high benefit of reperfusion strategy like systemic thrombolysis.

Importantly and in contrast to the aforementioned results, higher Mansoor's Self‐Report Tool for Cardiovascular Risk Assessment class was predictive for bleeding events in PE patients. High‐risk class according to Mansoor's Self‐Report Tool for Cardiovascular Risk Assessment was associated with 1.48‐fold risk of intracerebral bleeding, 1.32‐fold risk of gastro‐intestinal bleeding, and 1.17‐fold risk for the necessity regarding transfusion of blood constituents. Although several risk assessment models and scores have been developed in the past years to evaluate the individual bleeding risk,^21^, ^37^, ^39^, ^40^, ^41^, ^42^, ^43^, ^44^, ^45^, ^46^, ^47^, ^48^, ^49^ the Mansoor's Self‐Report Tool for Cardiovascular Risk Assessment is a very simple and easily to calculated score consisting of the patient characteristics age, sex, and existing comorbidities at the admission of the patient.^22^

Notably, prevalence of deep venous thrombosis and/or thrombophlebitis of the superficial veins was lower in high‐risk class of the Mansoor's Self‐Report Tool for Cardiovascular Risk Assessment. This finding is in line with some previously published study results, showing that deep venous thrombosis and/or thrombophlebitis of the superficial veins was related to lower case‐fatality rate in PE patients,^23^, ^50^, ^51^ whereas other studies revealed that concomitant DVT in PE patients was significantly associated with an increased risk of death within the first 30 days after symptomatic PE event.^52^ This discordance regarding short‐term case‐fatality of PE patients might indicate for the outstanding importance of the pathomechanism emphasizing embolization of the complete deep‐vein thrombus for patients with detected “isolated” PE.^51^ The relation between clot burden and PE patients' outcomes was highlighted in a few studies.^51^, ^52^, ^53^, ^54^

During the observational period, the total number of hospitalizations due to PE increased as previously reported.^7^ In total, approximately 1/3 of the PE patients was classified as high‐risk according to the Mansoor's self‐report tool for cardiovascular risk assessment, which seems proportionate. As expected, hospitalizations increased with patients' age and were largest in the 8th decade of life.^6^, ^7^, ^55^

Summarizing the results of our present study, the Mansoor's Self‐Report Tool for Cardiovascular Risk Assessment revealed only a weak prognostic performance to predict acute adverse in‐hospital events in PE patients in comparison to the sPESI. However, prognostic performance of the Mansoor's Self‐Report Tool for Cardiovascular Risk Assessment to predict bleeding events was after all moderate.

## 5 LIMITATIONS

There are some important limitations of our study that require main consideration: First, the study results of the present study are based on ICD‐ and OPS‐ discharge codes of patients hospitalized in German hospitals. This might lead to incomplete data based on under‐reporting or also under‐coding. Second, data about administration of most medical treatment such as anticoagularion treatment are not available in this dataset of the Federal Statistical Office of Germany. Third, we are not able to report long‐term (follow‐up) data, since the data are limited for analyzing the in‐hospital course of the patients only. Fourth, the exact cause of death could not be assessed since it is not coded in the German nationwide inpatient sample.

## 6 CONCLUSIONS

The prognostic performance of the Mansoor's Self‐Report Tool for Cardiovascular Risk Assessment for risk stratification of PE patients was poor in comparison to the sPESI, and it was not able to identify those PE patients, who might benefit from systemic thrombolysis. However, the Mansoor's Self‐Report Tool for Cardiovascular Risk Assessment was moderately helpful to identify PE patients at higher risk for bleeding events.

## CONFLICT OF INTEREST

KK, VHS, MAO, TM and CE‐K reported no conflict of interest. LH reports having received lecture honoraria from MSD. TM is PI of the DZHK (German Center for Cardiovascular Research), Partner Site Rhine‐Main, Mainz, Germany.

## ETHICS STATEMENT

Since in our present study the investigators had not a direct access to data of individual patients and the cumulative results were provided by the Research data center [RDC] of the Federal Statistical Office and the Statistical Offices of the federal states, approval by an ethics committee and informed consent were not required, in accordance with the German law.

## AUTHOR CONTRIBUTIONS

KK and LH contributed to the conception and were responsible for statistical analyses. KK wrote the first draft of the manuscript. All authors interpreted the data and critically reviewed the manuscript and approved the final version of the manuscript.

## Data Availability

The data that support the findings of this study are available from RDC of the Federal Statistical Office of Germany. Restrictions apply to the availability of these data, which were used under license for this study. Data are available from the author(s) with the permission of RDC of the Federal Statistical Office of Germany.
